# Draft genome sequence of a mosquito repellent *Bacillus licheniformis* strain Ba1 isolated from desert soil

**DOI:** 10.1128/MRA.00916-23

**Published:** 2023-11-15

**Authors:** Taha Chouati, Soufiane Maski, Marouane Melloul, Mohammed Ajdig, Lahcen Ouchari, Bahia Rached, Elmostafa El Fahime

**Affiliations:** 1 Genopath Research center, ERNN, Faculty of Medicine and Pharmacy, University Mohammed V in Rabat, Rabat, Morocco; 2 Supporting Unit for Scientific and Technical Research, National Center for Scientific and Technical Research, Rabat, Morocco; 3 Département de Biologie, Faculté des Sciences, Université Mohammed V, Rabat, Morocco; 4 Microbiology and Molecular Biology Team, Biodiversity and Environment Center, Faculty of Sciences, Mohammed V University, Rabat, Morocco; 5 Plant and Microbial Biotechnology, Biodiversity and Environment Center, Faculty of Sciences, Mohammed V University, Rabat, Morocco; 6 Laboratory of physical chemistry and biotechnologies of biomolecules and materials, University Hassan II Casablanca, FSTM, Mohammedia, Morocco; Indiana University, Bloomington, Bloomington, Indiana, USA

**Keywords:** *Bacillus licheniformis*, volatile organic compounds, whole genome sequencing, mosquitoes

## Abstract

Microbial volatile organic compounds have been shown to affect a wide insect behavior. In this paper, we report the draft genome sequence of *Bacillus licheniformis* strain Ba1 previously isolated from desert soil in Morocco. The assembled and annotated draft genome contains 4,726 coding genes, 6 rRNAs and 97 tRNAs.

## ANNOUNCEMENT

*Bacillus licheniformis* is a Gram-positive, endospore-forming organism with a nearly ubiquitous distribution in the environment (1). Its versatile and expanding industrial applications are well-recognized.

Microbial volatile organic compounds (mVOCs), which are carbon-containing compounds of low molecular weight generated during microbial metabolism, have been shown to influence the behavior of numerous insects, albeit the complete extent of these interactions remains to be fully understood (2).

Remarkably, while mVOCs have been studied extensively for their impact on various insects, there is limited information regarding repellent mVOCs targeting two significant disease vectors, *Anopheles gambiae* and *Aedes aegypti*, specifically from the Bacillus genus (3).

The studied strain was initially isolated from desert soil near Merzouga, a small Saharan Village in southeast Morocco (4). The isolation process involved homogenizing a 15 g sand sample in 15 mL of sterile saline solution (0.9% NaCl w/v), followed by 10-fold dilutions and plating on tryptone soy agar (TSA) (Difco, Detroit, USA) for 96 h at 55°C. Subsequently, fresh cultures were prepared on tryptone soy broth (TSB) at 37°C and 120 rpm agitation. Strain Ba1 was present in a mixture of strains whose VOCs demonstrated repulsive effects on both *Anopheles gambiae* and *Aedes aegypti* through volatile organic compounds (VOCs) (5).

Genomic DNA was extracted using an optimized protocol with a mechanical lysis step first (Precellys, two 30 s cycles at 6,500 rpm) and an enzymatic purification (Promega DNA Purification Kit). 16S rDNA identification was performed using universal primers Fdr (5′-AGAATTTGATCTTGGTTCAG-3′) and Rdm (5′-ACGGCTACCTTGTTACGACTT-3′) following MyTaq PCR kit instructions (Meridian Biosciences, OH, USA). The sequencing steps were performed using ExoSAP-IT, BigDye Terminator v3.1, and BigDye Xterminator (Thermo-fisher Scientific, MA, USA) according to standard protocol. The strain showed 99.98% nucleotide identity with *Bacillus licheniformis*.

Libraries were prepared following the instructions of Ion Xpress Plus gDNA Fragment Library Preparation kit purified (AMPure XP Beads) and size-selected for 200 bp reads (Thermo-fisher Scientific, MA, USA). Whole genome sequencing was conducted using ION Proton and ION Chef technologies and following the manufacturer’s instructions (Thermo-fisher Scientific, MA, USA).

**Fig 1:**
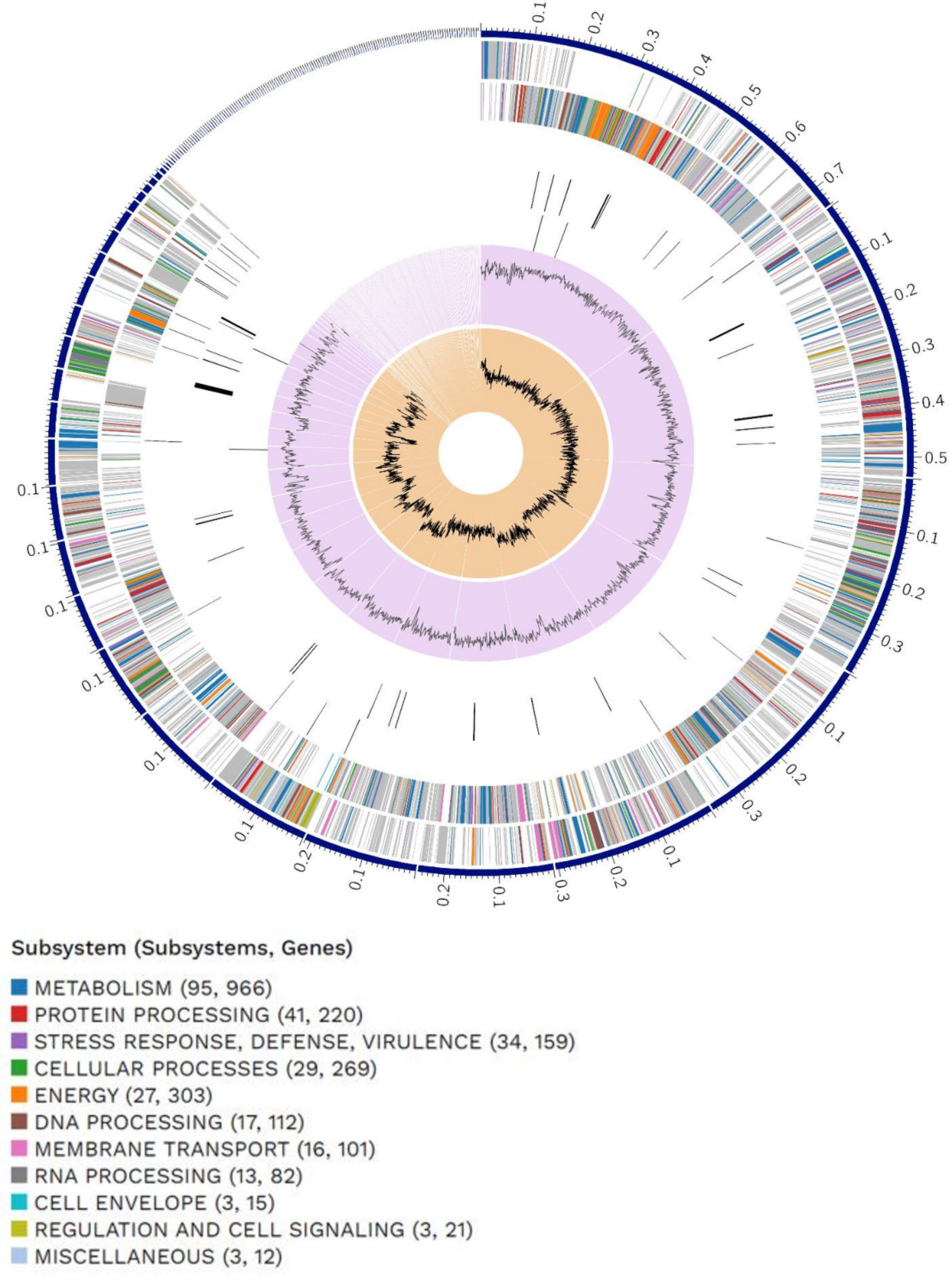
The distribution of genomic annotations is visually shown by a circular graphical display in PATRIC v3.26.4. The components included within this structure, progressing from the outermost to the innermost rings, consist of contigs, coding sequences (CDS) located on the forward strand, CDS located on the reverse strand, RNA genes, CDS exhibiting homology to established antimicrobial resistance genes, CDS exhibiting homology to recognized virulence factors, as well as GC content and GC skew. The chromatic attributes of the coding sequences on both the forward and reverse strands serve as indicators of the specific subsystem to which these genes are affiliated, as per the provided list.

Preliminary raw data (45160817 reads with an average length of 152) analysis and assessment wwere performed by the Ion Torrent Suite software v5.0.5 (ThermoFisher Scientific, MA, USA) at default parameters, followed by quality control on the Galaxy Platform (6) using FASTx quality statistics v1.0.1. Assembly was accomplished using SPAdes tool v3.13.1 (7) with k-mer length of 21,33,55,77,99, with the parameter –careful and assembly quality was assessed with QUAST v5.0.0 (8). Annotation was performed using NCBI Prokaryotic Genome Annotation Pipeline (PGAP) (9), followed by functional categorization using RAST v2.0 (10). Visualization of the of the genome was done using PATRIC v3.26.4 (11) (Fig. 1).

As shown in Table 1, the reconstructed genome of Ba1 is 4,227,162 bp long across 171 contigs. It has a GC content of 46.37% and an N_50_ of 305,417. The draft genome contains a total of 4,985 predicted genes of which 4,726 are CDS, 6 are rRNAs, and 97 are tRNAs.

**TABLE 1:** Assembly details^*a*^

Parameter	Value
Contigs	171
GC content	46.37%
Plasmids	0
Contig L50	5
Genome length	4,227,162 bp
Contig N50	305,417

^*a*^ An assembled genome was submitted to the Comprehensive Genome Analysis service. This assembled genome had 171 contigs, with the total length of 4,227,162 bp and an average GC content of 46.03%.

Secondary metabolites biosynthesis gene cluster analysis using antiSMASH v5.2 (12) has shown that Ba1 strain is predicted to be able to produce, among others, terpenes, type III Polyketides Synthases, thiopeptides and lanthipeptides, lassopeptides and non-ribosomal peptide synthetase cluster.

The analysis of the whole genome of the mosquito repellent strain of *Bacillus licheniformis* is an important step in identifying the genetic factors behind such effect.

## Data Availability

The strain sequence was submitted to NCBI under the Bioproject number PRJNA918595, the assembly is available under the accession GCA_029995335.1, the annotated sequences and raw reads are accessible under the accessions SRR22985052 and SRX18941248, respectively.
